# Early Onset Post-transplant Lymphoproliferative Disorder Presenting with Diarrhea Post-orthotopic Liver Transplant Treated Successfully with Single Rituximab Agent

**DOI:** 10.7759/cureus.6200

**Published:** 2019-11-19

**Authors:** Bonnie Patek, Cristina Strahotin

**Affiliations:** 1 Gastroenterology and Hepatology, Allegheny Health Network, Pittsburgh, USA

**Keywords:** post-transplant lymphoproliferative disorder (ptld), orthotopic liver transplant (olt), epstein-barr virus (ebv), rituximab

## Abstract

Post-transplant lymphoproliferative disorder (PTLD) is a rare complication seen in hematologic stem cell (HSC) and solid organ transplantation that results from immune suppressant medications needed to prevent allograft rejection. Epstein-Barr virus (EBV) has been implicated in a majority of these cases, specifically with B-cell-predominant lymphomas. We present a 57-year-old female who underwent an orthotopic liver transplant and presented with diarrhea and weight loss. At the time of transplantation, the patient's quantitative EBV titers were negative; however, repeat titers during her admission were positive. Infectious etiologies for diarrhea were negative so a colonoscopy was pursued which revealed large ulcerated areas and biopsies consistent with monomorphic, diffuse large B-cell lymphoma, plus EBV. Imaging revealed multiple areas below the diaphragm of lymphadenopathy. The patient was started on rituximab and antivirals, and immune suppressive medications were decreased with a resolution of her symptoms. PTLD after any transplantation can be difficult to diagnose, given the wide range of presenting symptoms. Identifying patients who are at high risk for developing PTLD may lead to a more timely diagnosis to initiate treatment and decrease mortality risk.

## Introduction

Post-transplant lymphoproliferative disorder (PTLD) is a rare complication of hematologic stem cell (HSC) and solid organ transplantation as a result of immunosuppressant medications that are necessary for the prevention of allograft rejection [1-2]. There have been several proposed risk factors that are associated with development of PTLD, including the patient's Epstein-Barr virus (EBV) status at time of transplant, the type of transplanted organ, the age at time of transplant, the duration and type of immunosuppressant medications that are used, and the underlying reason for transplant (notably, in the case of liver transplants) [2-5]. Pediatric transplant recipients have a higher rate of PTLD development at close to 10% compared to the adult transplant population of 2% - 3% [4]. Based on the organ transplanted, cardiac has the highest risk at 5%, followed by lung at 3.2%, liver at 2.8%, HSC at 1.7%, and renal at 1.5%; higher rates are likely related to the need for higher immune suppression to prevent rejection, allowing for replication of EBV in the setting of EBV-positive PTLD cases [1-2].

EBV status is an important component in the development of PTLD following liver transplantation. There is a higher association with EBV and early-onset PTLD (defined as within the first year of transplantation) and non-Hodgkins, mostly B-cell lymphomas [2, 6]. This subgroup of PTLD tends to be less aggressive compared to EBV-negative PTLD, which generally occurs three to five years post-transplantation [2, 6].

The clinical presentation of PTLD can be difficult to appreciate as it will range from asymptomatic patients to classic mononucleosis syndrome, multi-organ failure, or with symptoms only related to the associated extranodal involvement, which will vary depending on the organ(s) affected with extranodal invasion occurring 62% - 79% of the time [2, 7]. The gastrointestinal (GI) tract can be involved 23% - 56% of the time with symptoms ranging from iron deficiency anemia, failure to thrive, GI bleeding, perforation, intussusception, obstruction, or diarrhea [1, 7-8]. There are no standardized treatment regimens, given the rarity of this disease and lack of prospective randomized controlled trials; however, treatments are tailored around decreasing the dose of immunosuppressant agents. We present a case of PTLD following an orthotopic liver transplant (OLT) with presenting symptoms of diarrhea that was successfully treated with a single rituximab agent and decreased immunosuppression.

## Case presentation

A 57-year-old female with a history of alcoholic cirrhosis and heterozygosity for hemochromatosis underwent OLT four months prior with an uncomplicated postoperative course presented to the hospital for complaints of watery diarrhea and nausea for over one month with low-grade fevers.

Bowel movements were non-bloody, loose/watery, five times a day without associated abdominal pain. There were no flu-like symptoms, no sick contacts, sore throat, or noticeable lymphadenopathy except for the low-grade fever. The patient did endorse a 6-pound weight loss since diarrhea had started. Before being hospitalized, her symptoms were felt to be related to medication side effects so the tacrolimus was decreased to 1 mg po bid with a normal hepatic function panel and the mycophenolate was completely stopped without resolution of her symptoms.

Infectious disease workup was completed and was negative for Clostridioides difficile (C. diff), Yersinia, Cryptosporidium, Vibrio, ova and parasites, and rotavirus. Because of continued symptoms without infectious etiology, she had computed tomography (CT) of the abdomen/pelvis that showed non-specific enterocolitis and enlarged lymphadenopathy in the right upper quadrant, retroperitoneal region, and left-sided pelvic wall. EBV titers were checked and were 6,100 copies/ml; cytomegalovirus (CMV) deoxyribonucleic acid (DNA) was not detected. Importantly, the patient was CMV-positive/EBV-negative preoperatively and the donor was CMV-positive/EBV-positive.

**Figure 1:**
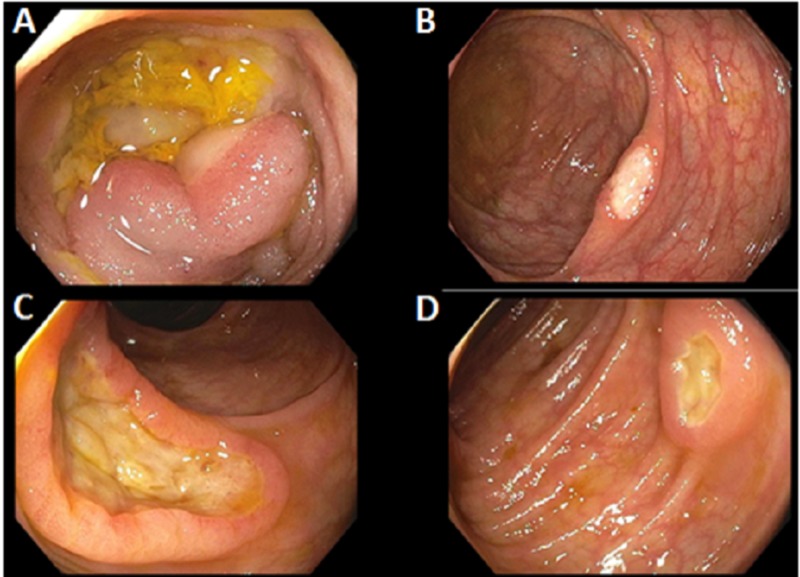
Initial colonoscopy A colonoscopy (indicated in the setting of non-infectious diarrhea in a post-orthotopic liver transplant patient) revealed multiple ulcerated areas within the terminal ileum (A) and throughout the entire colon (B-D).

A colonoscopy was completed showing multiple 1-3 cm deep, serpiginous, clean based ulcers throughout the entire colon and distal terminal ileum (Figure 1). The patient was started on ganciclovir based on imaging, the elevated EBV titers (peaked at 59,000 copies/ml), and colonoscopy results.

**Figure 2:**
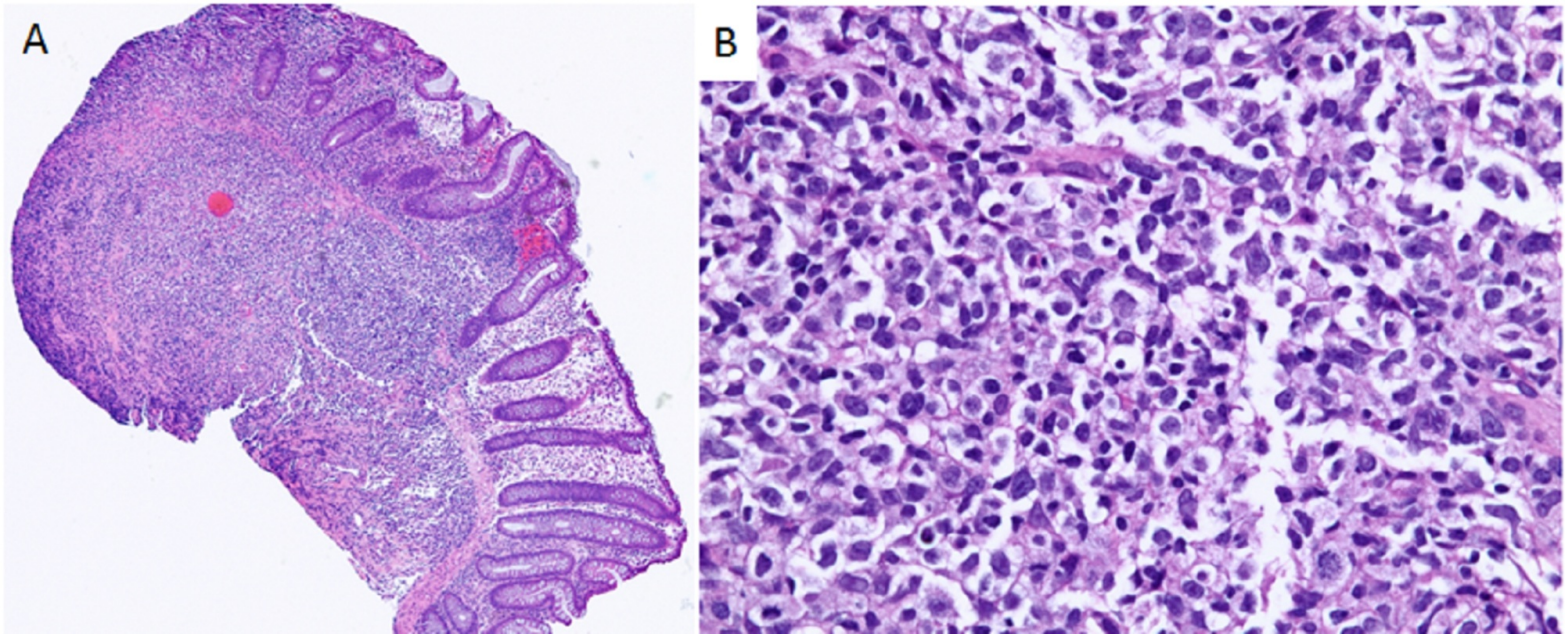
Histologic slides revealing atypical lymphoid infiltrate involving the colonic mucosa Additional immunostains demonstrated +CD20, CD79, BCL-2, CD30, and MUM-1 with in situ hybridizations positive for EBV RNA and negative for CMV or HSV (not pictured). (A) Low power; (B) 400x magnification. BCL-2: B-cell lymphocytic leukemia/lymphoma 2; CD: cluster of differentiation; CMV: cytomegalovirus; EBV: Epstein-Barr virus; HSV: herpes simplex virus; MUM-1: multiple myeloma oncogene-1; RNA: ribonucleic acid

Biopsies from the ulcers revealed monomorphic type, diffuse large B-cell lymphoma (DLBCL) non-germinal center type, plus EBV (Figure 2), at which time the patient was started on rituximab, 650 mg infusion weekly. Her treatment course was complicated by massive, obscure upper GI bleeding and she underwent interventional radiology embolization of a small proximal jejunal branch with active extravasation at the time of the intervention. The patient stabilized and was discharged without further complications and with complete resolution of diarrhea. A repeat colonoscopy two months later showed only one area of ulcerated mucosa with biopsies not concerning for histological evidence of lymphoma after receiving a total of eight cycles of rituximab.

## Discussion

PTLD is a rare complication post-solid organ transplant when compared with hematologic stem cell transplants that can present with several different symptoms. Post-OLT, the incidence is between 1% - 3% in adults with a mortality ranging from 40% - 60% [1, 9]. Etiology of PTLD is thought to be related to the immunosuppression that is required to prevent allograft rejection and is notably associated with EBV but can be EBV-negative which more often occurs late post-transplant, defined as over 12 months post-transplant and with a worse prognosis [4-6]. EBV-positive PTLD is more common with early-onset disease. PTLD tends to be B-cell lymphomas (85%) with the remaining cases being T-cell, natural killer cell, Burkitt’s lymphoma, or Hodgkin’s lymphoma [5-6]. PTLD, specifically B-cell lymphomas, tend to have an increased incidence of EBV positivity. One hypothesis for this is EBV DNA incorporates within B-cells post-initial infection where the virus remains dormant until immunosuppressant medications suppress CD8 T-cell support, leading to EBV replication without regulation [6]. The reactivation in the case of latent EBV can come from either the donor or the recipient's B-cells as there is lymphoid tissue within the transplanted liver [2-3]. Often, the recipient B-cells are the ones to proliferate in later cases, whereas the early onset cases that can typically affect the graft can be caused by the proliferation of the donor B-cells. In this case, the graft may have developed necrosis of the liver hilum, leading to compression of the portal hepatitis and then leading to further complications (like biliary stricture formation and portal vein thrombosis) which can be confused for rejection of the graft [9].

Risk factors for acquiring PTLD include the EBV status of the donor and recipient (highest risk in recipient-negative, donor-positive cases), type of organ that is transplanted, age at the time of transplant, and type of immunosuppression [2, 6]. Children tend to have higher rates of EBV-positive PTLD; this is thought to be related to a larger population that has not yet been exposed to the virus pretransplant and that with primary EBV infection viral loads tend to be higher [4]. The type of immune suppression that results in higher rates of PTLD is the calcineurin inhibitors or Thymoglobulin®, if needed, in the setting of rejection given the T-cell suppression [2]. mTOR inhibitors, such as sirolimus or everolimus, have been shown to decrease the risk of PTLD development with antiproliferative properties, and some patients benefit from switching from a calcineurin inhibitor to an mTOR inhibitor in the setting of PTLD [2-3, 5]. No increased risk has been associated with the use of inosine monophosphate dehydrogenase inhibitors like mycophenolate [2].

The development of PTLD can occur as early as 20 days to decades post-OLT [5]. The B-cell PTLD that is more commonly driven by EBV involvement can either be monoclonal or polyclonal, with monoclonal being more difficult to treat. Signs and symptoms of PTLD are vast and depend on whether it is limited to one organ, such as the allograft that typically occurs with early-onset PTLD (< 6 months post-transplant) or has a systemic involvement [2-3]. If a primary EBV infection occurs, signs and symptoms may include typical mononucleosis or a viral syndrome of pharyngitis with cervical lymphadenopathy, fatigue, headache, fever, malaise, and splenomegaly [1, 7]. If latent EBV in the setting of PTLD, lymphadenopathy is still common and may present anywhere in the body, such as mediastinal, mesenteric or retroperitoneal in addition to extranodal sites, including the stomach (12%), small bowel (15%), colon (6%), spleen (3%), liver (6%), lungs (3%) [5, 7]. Other atypical signs and symptoms that have been described include jaw pain, arthralgias, ascites, diarrhea, anemia, GI bleeding, GI tract obstruction or intussusception, and liver/periportal mass [7-8, 10]. There are no specific laboratory findings associated with PTLD as it varies depending on what organs are involved; however, an increased atypical lymphocytic predominant leukocytosis or abnormal elevation in liver function tests can be observed [1, 7]. Diagnosis is dependent on elevated levels of EBV DNA with tissue biopsy.

Treatment of EBV-positive PTLD is dependent on the type of PTLD. All treatment starts with decreasing the immunosuppression of the patient to allow for reactivation of T-cells, but this can come at a cost with possible graft rejection [9]. Some studies have shown successful treatment with changing immunosuppressant medications to mTOR agents, likely due to the antiproliferative effect that these medications have, in addition to immune suppression [2, 5]. Antiviral medications, such as ganciclovir/acyclovir, are typically started and may be of benefit if the PTLD is an EBV-positive primary infection/early stage (such as in polyclonal PTLD), as these agents inhibit lytic replication of EBV [9]. These medication mechanisms depend on an active viral thymidine kinase to convert the medication, but this thymidine kinase is often lacking or in such low quantities in latent EBV so that the PTLD tumors lack thymidine kinase, rendering the medication inactive and ineffective [9]. Foscarnet may be more effective by inhibiting viral DNA polymerase and some studies suggest that treatments aimed toward increasing lytic activity of the virus, such as activating the thymidine kinase, may make the tumors more susceptible to antiviral therapies and can be warranted in refractory cases [2, 11].

In the case of our patient, since her disease was monoclonal, antibodies against CD20 (B-cells) were utilized in addition to decreased immunosuppression. Treatment with anti-B-cell monoclonal antibodies (in this case, with rituximab) has been shown to improve prognosis overall - in one study up to 61% survival at three years [2]. Adverse side effects of rituximab should be observed in patients, specifically if GI-related, including perforation and obstruction [12]. In this case, GI bleeding occurred and may have been associated with the ulcerations within the colon, but this was unable to be confirmed with follow-up testing. If the patient’s response to rituximab is poor, the R-CHOP regimen (rituximab, cyclophosphamide, doxorubicin hydrochloride, vincristine (Oncovin), and prednisone) would be the next treatment option, but this is reserved for poor clinical response or more aggressive tumors in the setting of more severe potential toxic side effects [13]. Poor prognostic factors in the setting of PTLD include advanced age over 60 years, advanced disease stage based on the Ann Arbor staging scale, increased lactate dehydrogenase (LDH), extranodal disease, and poor functional status [2].

## Conclusions

The case presented herein is a unique presentation of PTLD. As described above, PTLD can present in many different ways with several diverse factors. Our case was within the early onset as the patient developed PTLD within about four months post-liver transplant, but her presenting symptom was diarrhea as opposed to primary graft involvement. This presenting symptom can complicate the clinical picture as there can be a delay in the diagnosis, given the multitude of reasons for a liver transplant patient to have diarrhea, including infection or medication-induced, which are more common explanations for diarrhea compared to PTLD and should be ruled out. The PTLD likely came from donor B-cell proliferation, given the EBV negativity in the patient before transplant, which is also interesting in that she presented without the involvement of the graft but rather extranodal disease in the setting of ulcerated colonic mucosa with extensive lymphadenopathy below the diaphragm. Once tissue diagnosis confirmed the diagnosis, single-agent anti-B-cell monoclonal antibodies were initiated in conjunction with the reduction of immunosuppressants. Antiviral medications in the setting of latent EBV and infection are unlikely to add additional therapeutic benefit. Given the primary findings of colonic ulcers, a repeat colonoscopy was warranted to evaluate a positive response to therapy, both endoscopically and histologically, in addition to repeat imaging. PTLD is a rare complication that can be difficult to diagnose initially as it can present with a magnitude of seemingly unrelated symptoms, but it should be pursued in patients post-liver transplant as other, more common etiologies must be investigated to prevent delay in treatments.
